# Scapular Morphology and Posterior Shoulder Stability: Biomechanical Evidence From an Advanced Cadaveric Shoulder Simulator

**DOI:** 10.1177/03635465251411312

**Published:** 2026-02-06

**Authors:** Bettina Hochreiter, Justine Fleurette, Mohammad Haddara, Bastian Sigrist, Richard Appleyard, Janos Tomka, Desmond Bokor, Matthias Zumstein, Sumit Raniga, Christian Gerber

**Affiliations:** † Department of Orthopaedics, Balgrist University Hospital, University of Zurich, Zurich, Switzerland; ‡ Macquarie University Translational Orthopaedic Research Lab (MQ TOR LAB), Faculty of Medicine, Health and Human Sciences, Macquarie University, Macquarie Park, NSW, Australia; § Research in Orthopaedic Computer Science (ROCS), Balgrist University Hospital, University of Zurich, Zurich, Switzerland; ‖ Shoulder, Elbow and Orthopaedic Sports Medicine, Orthopädie Sonnenhof, Bern, Switzerland; ¶ ResOrtho Foundation, Zurich, Switzerland; Investigation performed at the Macquarie University Translational Orthopaedic Research Lab, Macquarie Park, New South Wales, Australia

**Keywords:** biomechanics of bone, clinical medicine by anatomic region, clinical medicine by specialty interest, osteotomy, research (in vivo or in vitro), shoulder, general, shoulder, glenoid labrum, shoulder, instability

## Abstract

**Background:**

Static posterior shoulder subluxation (SPSL) is associated with both glenoid retroversion and altered acromial morphology. Although abnormal glenoid anatomy has been considered a crucial etiological factor, the biomechanical role of acromial anatomy remains incompletely understood.

**Hypothesis:**

Combined acromial and glenoid malalignment would produce greater posterior humeral head translation than either deformity alone, and targeted corrections could restore posterior stability.

**Study Design:**

Controlled laboratory study.

**Methods:**

Six fresh-frozen cadaveric shoulders underwent testing in a 6 degrees of freedom, 8-muscle actuated ex vivo cadaveric simulator. Seven conditions were tested: (1) intact, (2) posterior labral detachment, (3) isolated glenoid malalignment (–15° retroversion), (4) isolated acromial malalignment (high/flat), (5) combined malalignment, (6) acromial malalignment + glenoid correction + posterior acromial bone graft (PABG), (7) combined malalignment + PABG. Humeral head translation was measured during forward flexion at 30°, 50°, and 70° of elevation and normalized to glenoid width. Statistical analysis used repeated-measures analysis of variance with Bonferroni correction.

**Results:**

Posterior labral detachment showed minimal effect (1.3% ± 2.4% translation). On average, isolated glenoid malalignment increased posterior translation by 29%, whereas isolated acromial malalignment produced 31% posterior translation. Combined malalignment resulted in 54% posterior translation (*P* < .05 for all comparisons), demonstrating additive effects. Glenoid correction with PABG partially restored humeral head translation, but did not restore glenohumeral centering, with a residual 20% posterior translation compared with the intact shoulder. Adding a PABG to the combined malalignment led to a measurable reduction in posterior translation. However, although the graft decreased translation by approximately 13%, it did not restore native kinematics.

**Conclusions:**

Glenoid as well as acromial malalignment alone is associated with pathological posterior translation of the humeral head across the glenoid upon simulated active elevation. Combined acromial and glenoid malalignment produces significantly greater posterior translation than either deformity alone.

**Clinical Relevance:**

Complete anatomic correction of both deformities is necessary to restore normal posterior shoulder kinematics, supporting a comprehensive surgical approach for SPSL treatment.

Static posterior shoulder subluxation (SPSL) is a prearthritic condition defined by the permanent loss of glenohumeral concentricity with at least 55% of the humeral head posterior to the center of the glenoid, corresponding to a (glenohumeral) subluxation index >55% on axial computed tomography (CT) images.^26^ Static glenohumeral subluxation is almost invariably associated with a pathological so-called scapulohumeral subluxation index >55%, which is associated with glenohumeral subluxation alone, retroversion alone, or a combination of the 2 conditions.^8,27^ Over time, SPSL leads to eccentric posterior glenoid wear, described as Walch type B osteoarthritis (OA).^4,27^

The cause of this radiographically well-described entity remains incompletely understood: To date, most clinical and imaging studies have focused on glenoid morphology. Affected glenoids are typically more retroverted and inferiorly inclined compared with healthy shoulders, predisposing to posterior humeral head subluxation.^3,5,8,10,16,26^ A biomechanical study by Bryce et al^6^ demonstrated that a posterior bone defect of 5% in a B2 glenoid (equivalent to approximately 2.5° of glenoid retroversion) significantly increased posterior translation. Therefore, various proposed surgical techniques aim to correct glenoid morphology by opening osteotomy and/or bone grafting.^9,13,22,26,28^ Although these procedures often improve symptoms and correct retroversion, they do not consistently recenter the humeral head or halt the progression of eccentric wear.

Work by Beeler et al^2,3^ and Meyer et al^18,19^ showed that SPSL and eccentric OA can be associated not only with glenoid malalignment but also with acromial malalignment or, most frequently, a combination thereof. Indeed, those entities are associated with a higher, flatter, and shorter acromion in the sagittal plane. In addition, the acromion is significantly shorter in the frontal plane and even statistically significantly more so in SPSL than in dynamic posterior instability.^3^ This configuration results in a lack of posterior humeral head coverage and reduced resistance to posterior translation. Hochreiter et al^13,15^ demonstrated that correcting both acromial and glenoid morphology in 3D-printed as well as cadaveric models corrected excessive posterior humeral head translation and restored glenohumeral concentricity. A clinical case of SPSL treated with combined acromial and glenoid corrective osteotomies was reported by Gerber et al,^11,12^ resulting in a centered humeral head and resolution of symptoms at 2-year^12^ and 4-year^11^ follow-up. A pilot study by Gerber et al,^11^ furthermore, documented better recentering and restoration of clinical stability in 9 cases of recalcitrant posterior instability, confirming that the correction of the glenoid and acromial abnormalities found by Beeler et al^2,3^ could restore stability and improve centering of the glenohumeral joint in posterior instability associated with the documented skeletal variants.

In 1973, Scapinelli^23^ described a posterior acromial bone graft (PABG) technique using a scapular spine autograft to treat a patient with posterior shoulder subluxations. Scapinelli^24^ then published another article in 2006, using the same method in patients with dynamic posterior instability, with sustained success in 9 of 10 patients after 9.5 years. The effect of a PABG on posterior humeral head translation in the setting of normal glenoid morphology was recently described in 2 cadaveric studies.^14,25^ Testa et al^25^ and Hochreiter et al^14^ both demonstrated that PABG effectively restored posterior shoulder stability to near-native levels regardless of labral repair^25^ or in shoulders with acromial malalignment.^14^

The current study builds upon this body of work by examining the biomechanical influence of acromial and glenoid morphology on posterior humeral head translation, using an advanced, 6 degrees of freedom, cadaveric active motion shoulder simulator^1^ (Figure 1). The purpose of this study was to better understand how targeted acromial and glenoid corrections of defined deformities influence posterior translation of the humeral head during simulated active movements, influence centering of the glenohumeral joint, and therefore might have the potential to decelerate or even halt the development of eccentric OA.

**Figure 1.**
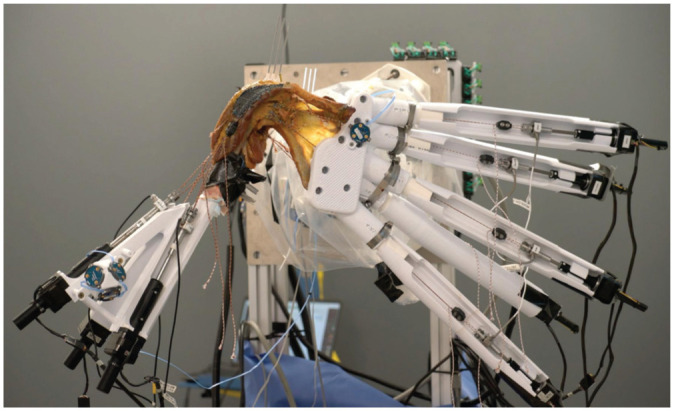
Shoulder simulator with all its components highlighted, including the linear actuators and their respective lines of action corresponding to specific muscles, optical markers for measuring humeral kinematics, and a 6 degrees of freedom Stewart platform for scapular motion.

We hypothesized that differences in scapular morphology would influence the translation of the humeral head. More precisely, a retroverted and downward tilted glenoid and/or a flatter and higher acromion would lead to an increase of posterior translation of the humeral head, potentially, resulting in abnormal kinematics with excessive posterior humeral head translation representing static and/or dynamic posterior shoulder subluxation.

## Methods

### Specimen Characteristics

**Figure 2.**
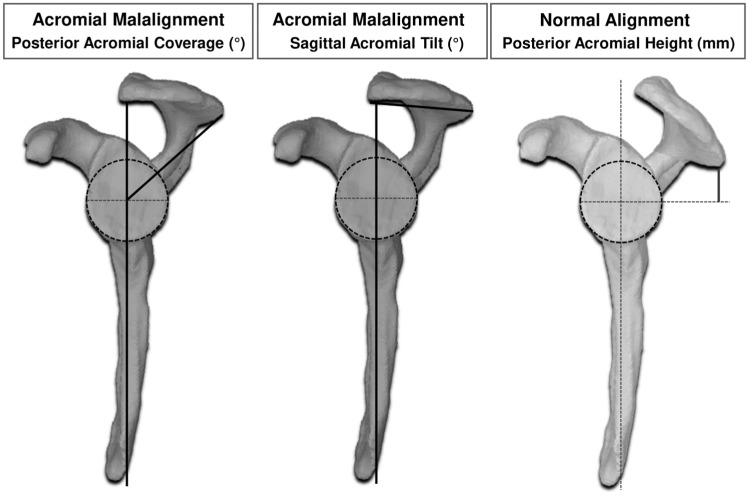
Illustration of (1) the anatomic differences between the 2 surgically achieved acromial conditions (adapted from Hochreiter et al^14^) and (2) acromial alignment measurements on segmented 3D models. The vertical dark gray lines correspond to the scapular plane. Bold lines correspond to specific measurements.

Six fresh-frozen cadaveric upper limbs (4 males, 2 females; mean age: 43.5 ± 8.7 years) were used in this study (sourced from ScienceCare). Ethical approval was obtained from the Macquarie University Human Ethics and Anatomy Governance Committee (Reference No. 52020606221936). Before inclusion, all specimens underwent CT imaging and were excluded if evidence of arthritis or other deficiencies were observed. Specimen selection was based on predefined inclusion criteria, which required specimens to be <60 years of age, along with additional morphological considerations outlined in Table 1. Overall, scapular morphology had to be normal in terms of glenoid and acromial morphology defined by Beeler et al.^3^ Table 2 presents the morphological characteristics of the included specimens. Preoperative 3D planning was conducted to achieve specific pathological values for posterior acromial height (PAH), posterior acromial coverage (PAC), sagittal acromial tilt (SAT), and glenoid version and inclination (Figure 2). The aim was to recreate the anatomic parameters (mean +1 standard deviation) seen in patients with SPSL.^3^ The surgically planned parameters for each specimen are detailed in Table 3.

**Table 1:** Morphological Inclusion Criteria and Normal Scapular Morphology

	Mean (Range)	Normal Values*^*a*^*
Posterior acromial height, mm	16.5 (11.8 to 20.6)	15.5 (4.9)
Posterior acromial coverage, deg	62.9 (54.5 to 73.5)	62.9 (7.5)
Sagittal acromial tilt, deg	58.2 (53.4 to 61.1)	55.7 (7.6)
Glenoid version, deg	−1.4 (–0.1 to −3.2)	−4.9 (4.9)
Glenoid inclination, deg	83.3 (77.4 to 88.9)	79.6 (4.3)
Critical shoulder angle, deg	31 (24.9 to 37.8)	31.7 (3.6)

a Normal values are according to Beeler et al.^3^

**Table 2:** Specimen Characteristics*^*a*^*

Specimen No.	Shoulder Side	Sex	Age, y	PAH, mm	PAC, deg	SAT, deg	Glenoid Version, deg	Glenoid Inclination, deg	CSA, deg
1	Left	Male	49	11.78	73.56	53.38	−2.21	77.36	32
2	Left	Male	33	18.62	61.56	61.06	−0.76	88.9	30.5
3	Right	Male	33	16.46	64.71	58.93	−0.54	88.5	32.4
4	Right	Female	43	15.52	54.57	59.5	−0.12	79.94	28.7
5	Right	Female	52	16.03	62.12	56.2	−3.15	83.67	37.8
6	Left	Male	51	20.55	61.39	60.26	−1.41	81.35	24.9

a CSA, critical shoulder angle; PAC, posterior acromial coverage; PAH, posterior acromial height; SAT, sagittal acromial tilt.

**Table 3:** Preoperative Surgical Plans*^*a*^*

	Planned Value	
	Specimens 1 and 6	Specimens 2, 3, 4, and 5
Posterior acromial height, mm	29.4	25
Posterior acromial coverage, deg	55	50
Sagittal acromial tilt, deg	80	80
Glenoid version, deg	−15	−15
Glenoid inclination, deg	85	85
Resulting Critical Shoulder Angle, deg	24.2 and 23.8	31.1, 35.3, 29.2, 38.1

a Specimen size variability necessitated different planning approaches. Planning a PAH of 29.4 mm was unfeasible in smaller specimens, creating unrealistic acromioglenoid relationships, whereas larger specimens with PAH 25 mm and SAT 80° resulted in acromiohumeral impingement. Therefore, 2 distinct configurations were required.

### Study Protocol

**Figure 3.**
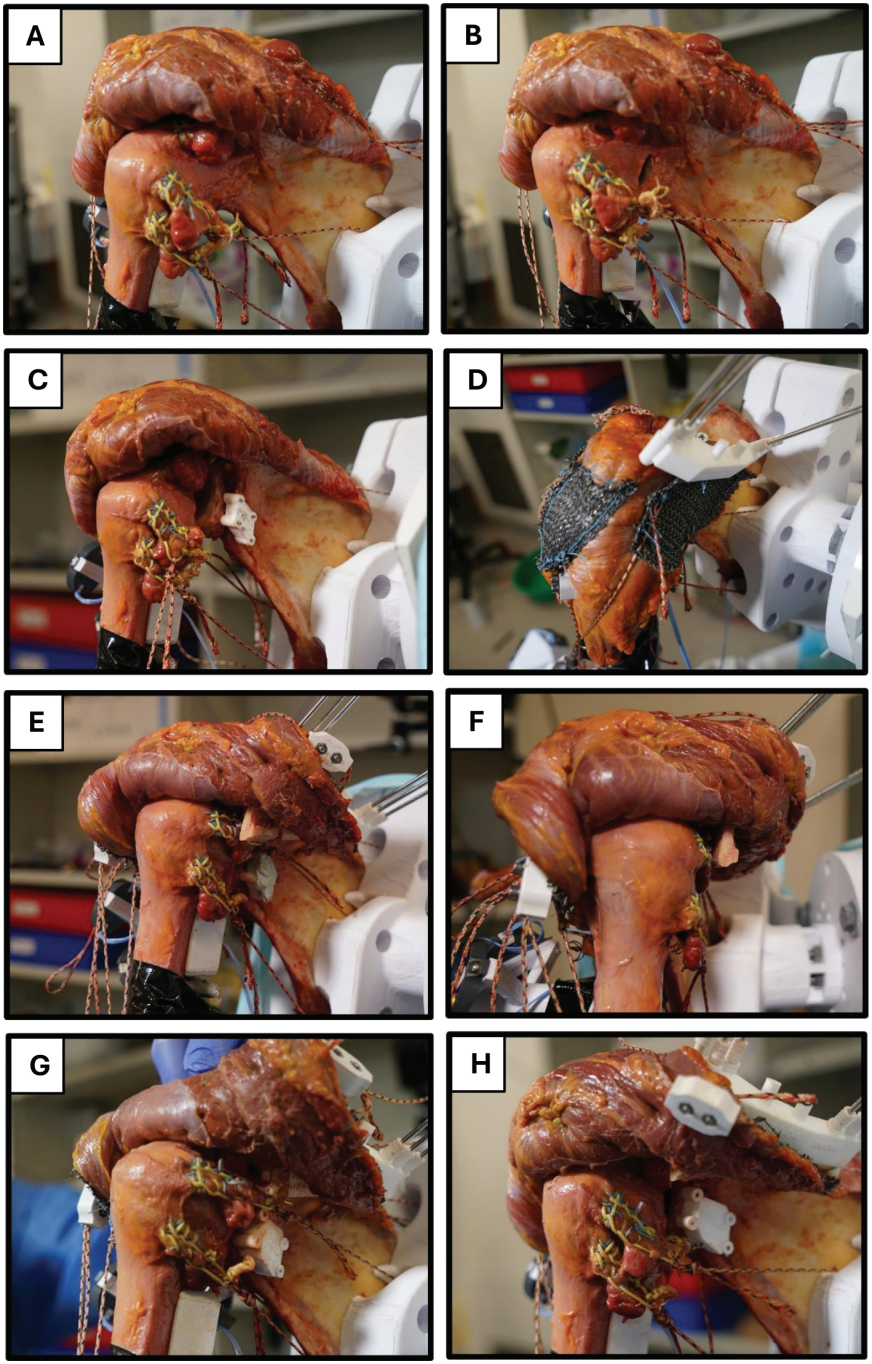
Surgical interventions and conditions that were investigated: (A) intact, (B) detached posterior labrum, (C) glenoid malalignment, (D) acromial malalignment, (E and F) acromial and glenoid malalignment + posterior acromial bone graft (PABG) (combined malalignment without PABG not shown), (G) acromial malalignment + glenoid correction, (H) acromial malalignment + glenoid correction + PABG.

**Figure 4.**
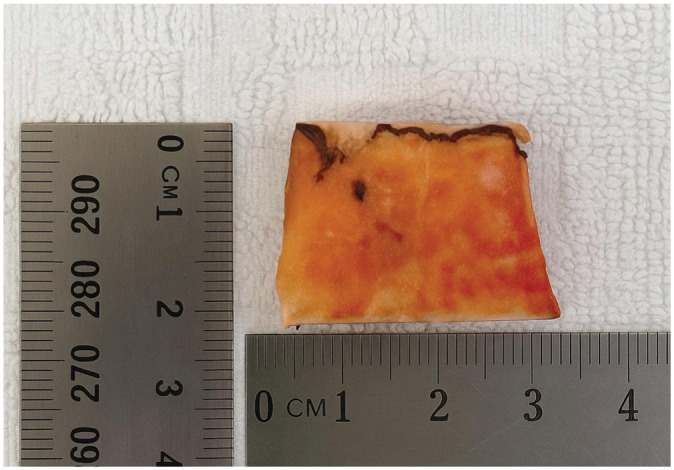
A 3 × 2.5–cm bone block was taken from the distal end of the humerus to form the desired posterior acromial bone graft.

To comprehensively investigate each surgical intervention and address the primary research question, a series of conditions were systematically tested (Figure 3). The study began with (1) the native intact glenohumeral joint (considered to also represent complete restoration of normal scapular anatomy) followed by (2) open surgical detachment of the posterior labrum (1 o'clock to 5 o'clock position) with injury of the posterior capsule. Subsequent conditions involved progressively invasive pathological simulations and, in the end, corrective procedures, including (3) glenoid malalignment (retroversion and downward tilt) and normal acromial alignment, (4) acromial malalignment (high and flat) and normal glenoid alignment, (5) combined acromial and glenoid malalignment, (6) the addition of a PABG (Figure 4) to the previous condition, and finally, (7) maintaining the acromial malalignment and PABG and correcting the glenoid. Glenoid and acromial malalignment was based on the mean values + 1 standard deviation of glenoid version, inclination, PAC, and PAH in Walch type B1 shoulders.^3^

Humeral head translation was recorded and evaluated under 2 distinct humeral motion pathways: (1) during forward flexion with maximal allowable internal rotation and (2) during forward flexion with maximal allowable external rotation.

### Creation of Glenoid and Acromial Malalignment and Glenoid Correction

#### Design and 3D Printing of Cutting and Reduction Guides

Segmented CT scans of all specimens were imported into CASPA (Computer Assisted Surgery Planning Application; Balgrist Campus, Zurich, Switzerland) to perform measurements of SAT, PAC, PAH, glenoid version, and inclination. Based on these values, individualized osteotomy planes and reductions were planned for each scenario (Table 3). For each specimen, personalized cutting and reduction guides for glenoid malalignment, acromial malalignment, and glenoid correction were designed in CASPA and 3D printed with PA12 using an SLS Printer (EOS Formiga P396).

### Surgical Technique for Creation of Scapular Malalignment and Correction

#### Glenoid Malalignment (Scenarios 3, 5, and 6)

Cutting guides were secured to the posterior glenoid and scapular neck using four 1.4-mm nonthreaded K-wires, resulting in excellent stability of the cutting guide on the bone. A posterior closing-wedge osteotomy was performed to create 15° of retroversion and 85° of inclination (Table 3) with the help of reduction guides that were then positioned over the preplaced K-wires to close the osteotomy and maintain the glenoid in its planned malalignment configuration (Figure 3, C-F).

#### Glenoid Correction (Scenarios 4 and 7)

For anatomic glenoid correction, the reduction guide used in scenarios 3, 5, and 6 was replaced with a second guide incorporating a predesigned wedge to open the osteotomy site and restore physiological glenoid version and inclination.

#### Acromial Malalignment (Scenarios 4-7)

**Figure 5.**
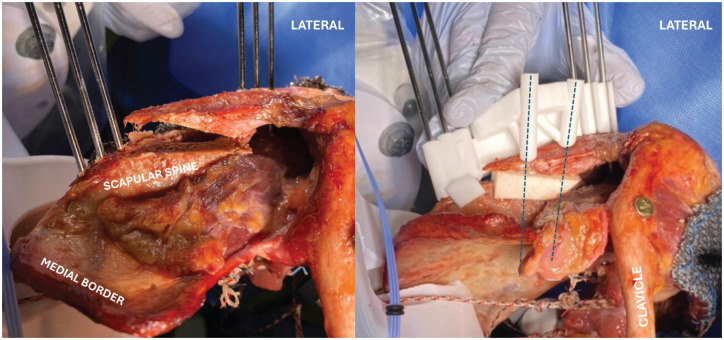
After osteotomy with cutting guides (on the left), a 3D-printed reduction block was placed between the medial and lateral acromial fragments to recreate the planned malalignment (on the right). Medial and lateral reduction guides were attached, and 2 preplanned 3.5 mm–diameter bicortical screws were inserted (dotted blue lines) to secure the lateral acromion and interposed block to the medial scapular spine. The reduction guides ensured that the position of the lateral acromial fragment was maintained during testing.

Cutting guides were fixed to the superior acromial surface and medial scapular spine with six 2.5-mm K-wires, resulting in excellent stability of the cutting guide on the bone. After osteotomy, a 3D-printed reduction block was placed between the medial and lateral acromial fragments (Figure 5) to recreate the planned malalignment (Table 3): PAH 25 to 29 mm, PAC 50° to 55°, and SAT 80°. Medial and lateral reduction guides were attached, and 2 preplanned 3.5 mm–diameter bicortical screws were inserted to secure the lateral acromion and interposed block to the medial scapular spine. The reduction guides ensured that the lateral acromial fragment was maintained in the intended position during testing. All guide blocks were designed to interface securely with bony anatomic landmarks and were fixed with the nonthreaded K-wires (Figure 3, C-H).

#### PABG (Scenario 6)

A 3.0 × 2.5–cm bone block (Figure 4) was harvested from the resected distal humeral shaft using an oscillating saw. The posterolateral acromial corner was exposed and the deltoid gently released from its insertion. The longest edge of the graft was positioned flush with the posterolateral acromial edge, and its undersurface was aligned parallel to the scapular plane, leading to an inferior overhang of the PABG resulting in a bony buttress according to the concept of Scapinelli (Figure 3, E and F).

The bony fragments were temporarily secured with two 1.6-mm K-wires. Two bicortical drill holes were created, and 3.5 mm–diameter cortical screws were inserted through the graft into the acromion. The deltoid was reattached over the graft construct.

### Specimen and Simulator Setup

#### Specimen Preparation

Before testing, all specimens were thawed at room temperature for approximately 26 hours. Shoulder dissection was meticulously performed to remove excess tissue, preserving the scapula, clavicle, and humerus along with the key soft tissues, which included an intact capsule, rotator cuff, and deltoid muscles. The muscle bellies of the supraspinatus, subscapularis, infraspinatus, and teres minor were excised, retaining approximately 5 cm of their tendinous insertions on the humeral head. Although the deltoid's origins on the scapula and clavicle were preserved, its humeral insertion was separated. The subscapularis was divided into superior and inferior parts, which were then actuated separately in accordance with Colin et al^7^ and Omi et al.^21^ The clavicle, detached from the sternum at the sternoclavicular joint, was secured to the coracoid process using a screw to ensure stability under forces exerted by the anterior deltoid.^1^

Preparations for connecting the rotator cuff and deltoids to their corresponding linear actuators involved multiple steps. Each rotator cuff tendon was reinforced with 2 layers of sutures. The first layer comprised a running locking Krackow stitch with a No. 2 OrthoCord or DynaCord braided composite suture (DePuy Synthes). The second layer involved another running locking Krackow stitch, this time using a braided Dacron fishing line (200-lb Tuf-Line Braided Dacron; O Mustad & Son). Subsequently, a braided Dacron line (9KM DWLife Braided Dacron Kite String, 200 lb) was attached to these sutures to facilitate the connection to actuators. Because the deltoid muscle bellies were left intact, a mesh patch (Coolaroo Heavy Duty Shade Cloth) was affixed to the lateral surface of each deltoid subregion using a running locking Krackow stitch with a No. 2 OrthoCord braided composite suture (DePuy Synthes). Finally, a Dacron line was secured to the patch to establish a connection between the deltoid muscles and the linear actuators.^1^

#### Anatomic Landmarks and 3D-Printed Mount System

To accommodate the unique anatomic structure of each shoulder specimen, custom 3D-printed specimen-specific mounts were designed for each scapula and humerus using individualized CT scans. These mounts were specifically developed to securely fix the scapula to the simulator base while preserving the native orientation of the bony anatomy. This ensured that the natural anatomic lines of action of the muscles were maintained during simulation, enabling physiologically relevant loading conditions and accurate biomechanical replication across specimens. The CT images of each specimen were carefully segmented to develop solid models of the scapula, clavicle, and humerus, which were then imported into SolidWorks (CAD) (Dassault Systèmes) for further refinement and analysis. Anatomic landmarks were defined as per standardized recommendations from the International Society of Biomechanics (ISB).^31^ Moreover, muscle origin and insertion points, as well as muscle lines of action, were labeled on the bone models. Each line of action was defined by connecting the origin and insertion points of the respective muscles to ensure that the actuators responsible for muscle loading applied forces along the true anatomic lines of action.

To ensure durability during simulation, all 3D models were printed in-house using Polylactic Acid + material with customized uniform infill density properties, providing adequate rigidity and strength for the mounts. This precise workflow facilitated accurate representation and functionality in simulating muscle and joint dynamics during testing.

#### Simulator Setup

Each specimen's 3D-printed mount, encompassing the shoulder girdle, was secured to a 6 degrees of freedom Stewart platform. The distal end of the humerus was resected using a bone saw to facilitate the attachment of the specimen-specific 3D-printed humeral mount.

A total of 8 actuators were used to apply controlled loading to the muscle groups of interest: 5 actuators (size 17; Picard Industries) for loading the rotator cuff muscles and 3 actuators (size 17; Picard Industries) dedicated to the 3 heads of the deltoid (Figure 1).

Joint and humeral kinematics were measured using an Optotrak Certus system (Northern Digital Inc), a high-precision 3-dimensional motion tracking system. Optical markers were affixed to the scapula mount and along the proximal humerus to capture humeral head translations and rotations during motion.

Each actuator was responsible for loading an individual muscle and continuously measuring in-line loads via uniaxial load transducers (Honeywell Model 31 Series) installed at the end of each actuator shaft. These load cells served as connectors between the sutured tendons/muscles and the corresponding actuator, ensuring precise force application and measurement.

#### Coordinate System Adjustments for Analyzing Humerus-to-Glenoid Motion

To accurately assess glenohumeral kinematics, humeral motion was initially captured relative to a scapular coordinate system defined according to ISB standards. Post hoc transformations were then applied to express humeral motion relative to the glenoid, using digitized anatomic landmarks and surgical planning data (Appendix, available in the online version of this article). These transformations accounted for individualized changes in glenoid version and inclination, as well as simulated acromial tilts, ensuring that humeral positioning was analyzed relative to the functional articular surface. This method enabled anatomically accurate and condition-specific motion analysis across specimens while preserving consistency with established biomechanical reference frames. The Appendix is available for a more detailed explanation of the transformation procedures.

### Statistical Analysis

Due to the lack of prior quantitative data on combined acromial and glenoid malalignment, an a priori power analysis could not be performed. Instead, a post hoc power analysis was conducted to evaluate the sensitivity of the observed data. Given the large within-specimen effect sizes and low variability in this repeated-measures design, the analysis indicated very high (>0.99) statistical power for detecting the primary biomechanical effects in humeral head translation. This reflects the strength of the observed effects rather than the adequacy of the initial sample size. The limited number of specimens reflects the substantial technical and surgical preparation required for each specimen and is acknowledged as a limitation of the study.

To normalize data to specimen size and specifically account for differences in glenoid size, humeral head translation (in millimeters) was reported as a percentage of individual glenoid width.

One-way repeated-measures analyses of variance, incorporating a Bonferroni correction, were conducted to assess the biomechanical effects of various pathological states and determine how subsequent surgical repairs influenced kinematic changes in humeral head translation. To account for variability among specimens and ensure robust statistical evaluation, within-subject effects were analyzed, allowing for direct comparisons to be made within the same specimen. Pairwise comparisons were also performed to identify specific differences between pathological states and their respective surgical corrections. A significance threshold of *P* < .05 was applied to all statistical tests.

## Results

**Figure 6.**
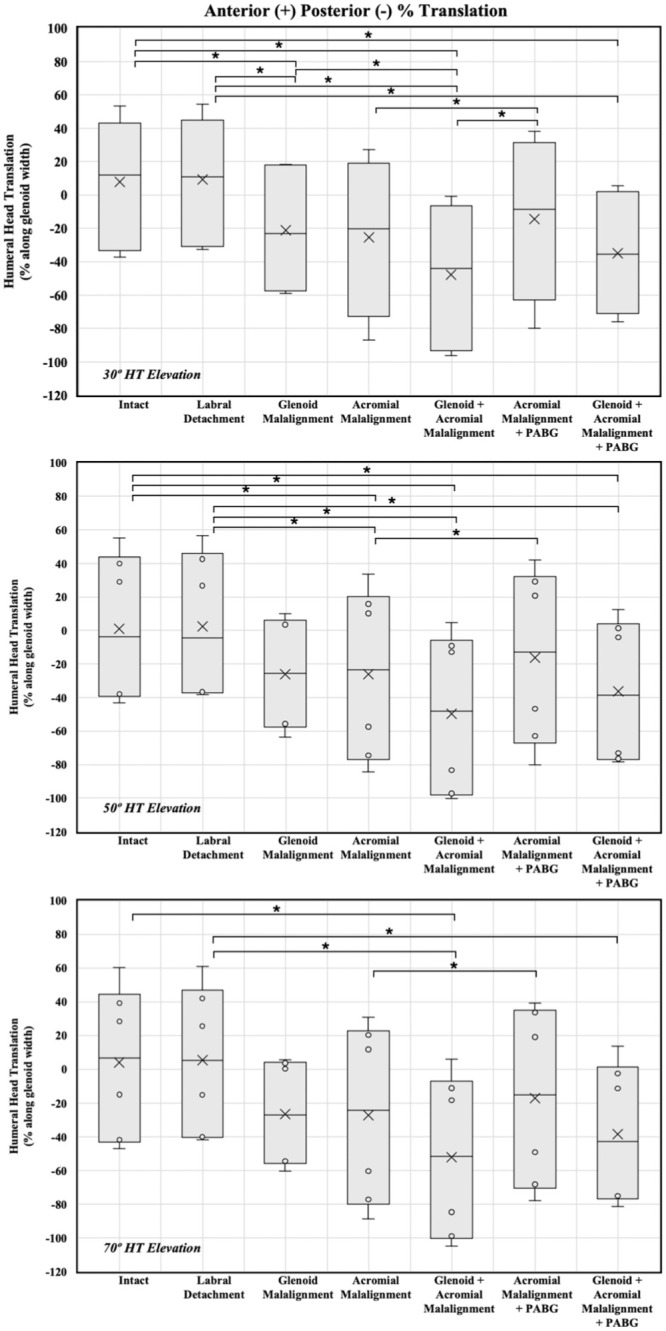
Within-specimen differences in humeral head translation (% along glenoid width) between tested scenarios at 30°, 50°, and 70° of humerothoracic elevation. Positive values correspond to posterior translation; negative values correspond to anterior translation. HT, humerothoracic; PABG, posterior acromial bone graft.

Results are shown in Table 4 and Figure 6. Across all specimens, detachment of the posterior labrum demonstrated minimal and statistically insignificant effects on humeral head translation with a mean anterior translation of 1.3% ± 2.4%, 1.3% ± 2.5%, and 1.3% ± 2.7% at 30°, 50°, and 70° of humerothoracic elevation, respectively, relative to the intact condition (*P*≥ .999 for all comparisons).

**Table 4:** Within-Specimen Differences in Humeral Head Translation (% Along Glenoid Width) Between Tested Scenarios*^*a*^*

		30° Elevation			50° Elevation			70° Elevation		
		Mean Δ	SD	*P*	Mean Δ	SD	*P*	Mean Δ	SD	*P*
Intact	Labral detachment	−1.3	2.4	≥.999	−1.3	2.5	≥.999	−1.3	2.7	≥.999
	Glenoid malalignment	29	9.2	**.012**	27.1	13.0	.079	30.6	18.3	.197
	Acromial malalignment	33.3	21.2	.252	27.3	11.1	**.038**	31.3	21.6	.349
	G + A malalignment	55.6	17.2	**.011**	50.9	13.0	**.004**	56	22.2	**.034**
	Acromial malalignment + PABG	22.4	23.1	≥.999	17.5	13.5	.523	21.2	21.5	≥.999
	G + A malalignment + PABG	42.9	14.6	**.017**	37.5	9.6	**.004**	42.4	18.7	.055
Glenoid malalignment	Intact	−29	9.2	**.012**	−27.1	13.0	.079	−30.6	18.3	.197
	Labral detachment	−30.2	8.2	**.006**	−28.4	12.8	.059	−31.8	17.5	.140
	Acromial malalignment	4.3	15.6	≥.999	0.2	20.7	≥.999	0.7	24.1	≥.999
	G + A malalignment	26.7	9.1	**.018**	23.8	14.2	.195	25.4	17.8	.366
	Acromial malalignment + PABG	−6.6	17.2	≥.999	−9.6	22.1	≥.999	−9.4	24.1	≥.999
	G + A malalignment + PABG	14.0	6.6	.073	10.4	9.4	.891	11.9	11.6	≥.999
Acromial malalignment	Intact	−33.3	21.2	.252	−27.2	11.1	**.038**	−31.3	21.6	.349
	Labral	−34.5	21.1	.213	−28.6	11.4	**.035**	−32.5	21.5	.294
	Glenoid malalignment	−4.3	15.6	≥.999	−0.2	20.7	≥.999	−0.7	24.1	≥.999
	G + A malalignment	22.4	13.0	.177	23.6	15.6	.294	24.7	17.8	.406
	Acromial malalignment + PABG	−10.9	2.3	**.002**	−9.8	3.2	**.014**	−10.1	2.2	**.002**
	G + A malalignment + PABG	9.7	14.5	≥.999	10.3	14.3	≥.999	11.2	19.5	≥.999
G + A malalignment	Intact	−55.6	17.2	**.011**	−50.9	13.0	**.004**	−56	22.2	**.034**
	Labral	−56.9	16.7	**.009**	−52.2	12.8	**.004**	−57.2	21.6	**.027**
	Glenoid malalignment	−26.7	9.1	**.018**	−23.8	14.2	.195	−25.4	17.8	.366
	Acromial malalignment	−22.4	13.0	.177	−23.6	15.6	.294	−24.7	17.8	.406
	Acromial malalignment + PABG	−33.3	13.5	.037	−33.4	15.7	.072	−34.8	17.6	.100
	G + A malalignment + PABG	−12.7	8.7	.328	−13.3	6.5	.082	−13.5	8.3	.214

a Positive values correspond to posterior translation; negative values correspond to anterior translation. Boldface indicates statistical significance. G + A, glenoid and acromial; PABG, posterior acromial bone graft.

### 30° of Humerothoracic Elevation

Compared with the intact condition, glenoid malalignment (–15°) induced a significant posterior humeral head translation of 29% ± 9.2% (*P* = .012), acromial malalignment alone (eg, glenoid corrected) showed a posterior translation of 33.3% ± 21.2%, (*P* = .252), and combined glenoid and acromial malalignment resulted in an increase in posterior translation of the humeral head of 55.6% ± 17.2% (*P* = .011).

Adding a PABG to acromial malalignment alone (eg, normal or corrected glenoid) resulted in a posterior translation of 22.4% ± 23.1% (*P*≥ .999 compared with intact), which was a significant decrease of 10.9% compared with acromial pathology alone (*P* = .002). Adding a PABG to the combined glenoid and acromial malalignment led to a posterior translation of 42.9% ± 14.6%, significantly more compared with the intact condition (*P* = .017) and an insignificant reduction of 12.7% (*P* = .328) compared with glenoid and acromial malalignment.

### 50° of Humerothoracic Elevation

At moderate elevation angles, compared with the intact condition, glenoid malalignment alone resulted in posterior humeral head translation of 27.1% ± 13% (*P* = .079), acromial malalignment alone led to a posterior translation of 27.3% ± 11.1% (*P* = .038), and combined glenoid and acromial malalignment resulted in a posterior translation of 50.9% ± 13% (*P* = .004), representing an increase of another 23.4% (*P* = .177) compared with acromial malalignment alone.

Adding a PABG to the isolated acromial deformity reduced the translation to 17.5% ± 13.5% (*P* = .523 compared with intact), a significant decrease of 9.8% compared with acromial malalignment alone (*P* = .014). Adding a PABG to the combined glenoid and acromial malalignment resulted in a posterior translation of 37.5% ± 9.6%, significantly more compared with the intact condition (*P* = .004) and an insignificant reduction of 13.3% ± 6.5% (*P* = .082) compared with glenoid and acromial malalignment.

### 70° of Humerothoracic Elevation

At a 70° elevation angle, compared with intact, glenoid malalignment alone increased posterior humeral head translation by 30.6% ± 18.3% (*P* = .197), acromial malalignment alone by 31.3% ± 21.6% (*P* = .349), and combined acromial and glenoid malalignment by 56% ± 22.2% (*P* = .034).

Adding a PABG to isolated acromial malalignment reduced posterior translation to 21.2% ± 21.5 (*P*≥ .999 compared with intact), a significant decrease of 10.1% ± 2.2% compared with acromial malalignment alone (*P* = .002). Adding a PABG to the combined glenoid and acromial malalignment resulted in a posterior translation of 42.4% ± 18.7% (*P* = .055 compared with intact) and an insignificant reduction of 13.5% ± 8.3% compared with glenoid and acromial malalignment (*P* = .214).

## Discussion

The most important findings of this study are that (1) both glenoid and acromial malalignment alone were associated with increased posterior humeral head translation upon simulated active elevation, (2) combined deformities led to substantially greater posterior translation than either one alone, and (3) in the setting of a combined pathology, anatomic correction—via either glenoid reorientation or reorientation plus PABG—could significantly reduce but not fully restore pathological posterior translation. Complete restoration of normal biomechanics, however, requires correction of both acromial and glenoid morphology to reestablish their normal anatomic relationship. These results underscore the complex role of scapular anatomy in SPSL and suggest that according to the degree of the pathological changes in glenoid and acromial anatomy, a targeted anatomic approach addressing both glenoid and acromial morphology may be necessary to restore posterior stability in affected patients.

These findings build on previous clinical and biomechanical evidence suggesting that scapular morphology, and not just glenoid version, contribute to SPSL and development of eccentric OA. Although glenoid retroversion has long been assumed to be the primary driver of posterior subluxation,^5,6,8,26^ more recent work has identified altered acromial morphology—namely, increased PAH, decreased PAC, and decreased SAT—as additional risk factors.^2,3,13,15^ Our data confirm and extend these observations, showing that when both glenoid and acromial pathoanatomies are present, posterior translation is significantly increased during elevation.

Interestingly, surgical detachment of the posterior capsulolabral complex did not increase posterior translation of the humeral head throughout the range of motion. This contrasts with previous biomechanical studies,^17,29,30^ which typically simulated posterior instability using a load-and-shift or jerk test and often reported a significant stabilizing role of the posterior labrum. The key difference lies in our simulation model. In the present study, the humerus was not translated posteriorly under axial load. Instead, motion was actively driven by rotator cuff and deltoid muscle forces, closely replicating physiological, functional movement patterns. It is important to understand that the simulator's control algorithm inherently operates by remapping muscle excursions to maintain humeral head centering. Therefore, our interpretation is that after posterior labral detachment, the balance of the rotator cuff was altered, leading the anterior muscles to increase their pull in an effort to center the head. This distinction suggests that although the posterior labrum may contribute to stability under direct, forced translation, it appears to have limited relevance in the pathogenesis of SPSL. It is interesting that in the pilot study of posterior instability in which acromial and scapular corrections were carried out, the labrum was never addressed or even repaired, and to date, recurrence of instability has not occurred in this published series or in >30 more recent cases.^11^ Clinically, eccentric OA is observed not only in young, athletic males subjected to repetitive loading but also in older individuals whose shoulders may never have experienced high axial loads in flexion and internal rotation. This pattern supports our findings and is compatible with observations of Moroder et al,^20^ who reported symptomatic improvement—but no radiographic recentering of the humeral head and progression of OA—after the arthroscopic posterior articular coverage and shift procedure. In that technique, the posterior capsulolabral complex is shifted anteriorly and fixed into the cartilage defect. These results refute a long-term stabilizing effect of the posterior labrum and a role in the development and persistence of SPSL. Our findings suggest that posterior shoulder stability is primarily anthropometrically driven, dependent on skeletal rather than on capsulolabral anatomy. Clinically, this supports a treatment strategy focused on correcting scapular anatomy necessitating either glenoid, acromial, or combined corrections. Consequently, a combined bony correction approach can serve as a biomechanical surrogate for the native joint, even in the absence of intact posterior capsulolabral structures, and may represent a joint-preserving alternative in the early management of SPSL.

Although not always statistically significant, both glenoid and acromial malalignment consistently resulted in increased posterior humeral head translation, ranging between 27% and 33% with the amount of malorientation of the acromion and/or glenoid selected in this study. This also demonstrates that glenoid correction alone is insufficient to recenter the humeral head in the presence of a high, flat acromion—an observation that aligns with findings from the clinical literature.^2,3,13,15,18,19^

The frequently reported increase of glenohumeral OA after open wedge osteotomies or posterior bone blocks may be related to the relatively short acromion typical for these cases.^3^ Although the critical shoulder angle (CSA) has never been reported, it may be necessary to look at this parameter in posterior shoulder instability, especially if early OA is present or procedures increasing joint pressure are envisioned.

The most pronounced subluxations occurred in the combined pathological condition, consistently exceeding 50% of glenoid width. This additive effect supports the notion that glenoid and acromial malalignment are not isolated contributors but act jointly to compromise joint stability in SPSL. Clinically, this degree of posterior translation during activities of daily living—particularly those performed below shoulder height—may plausibly contribute to the development and progression of eccentric OA.

Adding a PABG to the combined pathological state led to a measurable reduction in posterior translation. However, although the graft decreased translation by approximately 13%, it did not restore native kinematics. This is consistent with previous cadaveric work showing that a posterior acromial extension can significantly reduce translation,^25^ but acromial correction may not be sufficient in isolation.^15^ Except for full restoration of glenoid and acromial alignment (ie, intact condition), anatomic correction of glenoid retroversion and inclination in combination with a PABG most effectively restored humeral head centering, within approximately 20% of normal posterior translation. Clinically, correction of acromial and glenoid morphology to values of a statistical shape model of a normal shoulder has so far proven effective, but in a small series of glenoid corrections with the addition of a PABG the clinical results are also excellent, suggesting that the amount of correction in an individual case to restore shoulder function is not yet known.^11^ The early findings of PABG associated with normally oriented glenoids suggest that the graft may also be a useful adjunct in early-stage static posterior instability, especially if the lateral extension of the acromion is not pathologically short (small CSA).

Although we did not formally test the combined correction of both glenoid and acromial morphology, it is reasonable to assume that, provided it is realized successfully, such a scenario approximates the native condition most closely. None of the tested interventions restored normal kinematics, with the combination of glenoid correction plus PABG coming closest. It may well be that this combination is a laudable alternative to the combined correction of acromial and glenoid malalignment as suggested with the SCOPE osteotomy.^11,12^

In patients with SPSL or early-stage eccentric OA and minimal glenoid wear, anatomically tailored correction of scapular malalignment may offer a joint-preserving strategy to restore posterior stability and potentially delay or prevent disease progression. Early results of Gerber et al^11,12^ with combined correction of glenoid version and acromial malalignment with the SCOPE procedure (scapular corrective osteotomies for posterior escape) show at least the potential for midterm restoration of static and dynamic instability. Future studies should aim to validate this concept clinically and establish morphological thresholds to guide decision making—specifically, when isolated glenoid or acromial correction may be sufficient and when a combined approach is indicated.

This study has several limitations. One of the most important limitations is that only 1 defined set of changes was tested (not 5°, 10°, 15°, etc, of glenoid retroversion, and not many variants of acromial malformation/malalignment), which limits applicability of the data in a clinical setting. Therefore, it is a proof-of-concept study showing that only complete restoration of the anthropometric relations between glenoid and acromion restores glenohumeral kinematics. How much deviation from normal can be tolerated or be compensated for could not be tested. The complex interplay of static and dynamic stabilizers in the shoulder cannot be fully reproduced in a cadaveric model. Additionally, the bone and soft tissue quality of the specimens may not accurately reflect that of living patients. The multiple testing conditions and repeated loading cycles could have introduced progressive soft tissue degradation. To mitigate this risk, the testing sequence was structured such that the malaligned acromial conditions were assessed early—after the native state and before any corrective procedures—when the soft tissues were likely in their most intact and stable state. This approach was intended to reduce the impact of tissue fatigue and preserve soft tissue integrity during subsequent testing of corrected configurations. Initially, the PABG was intended to be placed—as described by Scapinelli^23,24^ and implemented in previous biomechanical studies^25^—in slight contact with the infraspinatus tendon. Unfortunately, this was not feasible, as the actuators for the external rotators became entangled with the graft, rendering the simulator nonfunctional in that configuration. As a result, the position of the PABG was adjusted and ultimately aligned parallel to the scapular plane, which allowed for more posterior translation of the humeral head. Further optimization of graft positioning and size may lead to even greater improvements in stability. Finally, the PABG could not be harvested from the scapular spine as originally described, as this structure served as a landmark for guide positioning; however, clinically the authors generally prefer to use a bone graft from the iliac crest.^14^

## Conclusion

Isolated glenoid and/or isolated acromial malalignments are associated with markedly increased posterior translation of the humeral head upon experimental, active anterior elevation. Combined acromial and glenoid malalignment produces significantly greater posterior humeral head translation than either deformity alone. Complete anatomic correction of both deformities is necessary to restore normal posterior glenohumeral translation, supporting a comprehensive surgical approach for SPSL treatment.

## Supplemental Material

sj-docx-1-ajs-10.1177_03635465251411312 – Supplemental material for Scapular Morphology and Posterior Shoulder Stability: Biomechanical Evidence From an Advanced Cadaveric Shoulder Simulator Supplemental material, sj-docx-1-ajs-10.1177_03635465251411312 for Scapular Morphology and Posterior Shoulder Stability: Biomechanical Evidence From an Advanced Cadaveric Shoulder Simulator by Bettina Hochreiter, Justine Fleurette, Mohammad Haddara, Bastian Sigrist, Richard Appleyard, Janos Tomka, Desmond Bokor, Matthias Zumstein, Sumit Raniga and Christian Gerber in The American Journal of Sports Medicine
