# Supplementary material for: Scapular Morphology and Posterior Shoulder Stability: Biomechanical Evidence From an Advanced Cadaveric Shoulder Simulator
Source: Am J Sports Med. 2026 Feb 6;54(4):857–68. doi: 10.1177/03635465251411312 (PMC12949044; doi:10.1177/03635465251411312)
Supplement: sj-docx-1-ajs-10.1177_03635465251411312 – Supplemental material for Scapular Morphology and Posterior Shoulder Stability: Biomechanical Evidence From an Advanced Cadaveric Shoulder Simulator [file sj-docx-1-ajs-10.1177_03635465251411312.docx]

**Post-Hoc Kinematic Transformations for Humerus-to-Glenoid Motion Analysis**

In this study, humeral motion was initially recorded relative to the scapula using coordinate systems defined according to the International Society of Biomechanics (ISB). Landmarks were digitized with a calibrated stylus in a custom workspace within The MotionMonitor software.

- Scapular coordinate system (S): origin at the angulus acromialis (AA). The Z-axis was aligned from the trigonum spinae (TS) to AA, the Y-axis pointed superiorly from the inferior angle toward the midpoint between TS and AA, and the X-axis was orthogonal to these (pointing laterally in a right shoulder).
- Humeral coordinate system (H): origin at the humeral head center. The Y-axis was directed from the humeral head toward the midpoint of the epicondyles, the X-axis from lateral to medial epicondyle (pointing laterally in a right shoulder), and the Z-axis orthogonal to these.

For functional analysis of glenohumeral kinematics, humeral motion was re-expressed in a glenoid coordinate system (G), constructed after digitizing the articular surface. In this frame, the Y-axis was aligned superior–inferior, the X-axis posterior–anterior, and the Z-axis orthogonal to the glenoid face.

The transformation from the scapula-based frame (at AA) to the glenoid frame was computed as:

1. *P*_humerus∣glenoid_​ = (*R*_scapula∣glenoid_ ​⋅ *P*_humerus∣AA_​) + *P*_scapula∣glenoid_

where:
• *P*_humerus∣glenoid_ is the humerus position in the glenoid frame,
• *R*_scapula∣glenoid_ is the scapula orientation relative to the glenoid,
• *P*_humerus∣AA_​ is the humerus position relative to the scapular origin (AA),
• *P*_scapula∣glenoid​_ is the scapula origin (AA) expressed in the glenoid frame.

**Planned Surgical Adjustments (Active Rotations)**

To simulate surgical interventions, additional active rotations were applied to the scapula (and thus the glenoid). These included planned changes in glenoid version (about the Y-axis) and inclination (about the X-axis).

The updated scapula orientation relative to the glenoid was calculated as:
*(2) R_new scapula∣glenoid =_ R_X_ (θ_X_) ⋅ R_Y_ (θ_Y_) ⋅ R_scapula∣glenoid_ ​*
and the updated scapula position as:
*(3) P_new scapula∣glenoid =_ R_X_ (θ_X_) ⋅ R_Y_ (θ_Y_) ⋅ P_scapula∣glenoid_ ​*

These updates correspond to physically tilting the glenoid about its own axes, pivoting at the glenoid origin. The new humerus position in the glenoid frame then followed directly:

*(4) P_new humerus∣glenoid_​ = (R _new scapula∣glenoid_ ​⋅ P_humerus∣AA_​) + P_new scapula∣glenoid​_*

**Adjustments to the AA Frame**

In some conditions, the AA-based scapular frame itself was adjusted. This was implemented as a rotation about the Z-axis by θ_Z_, followed by a translation along the Y-axis by TY:
 (5) *P*_new humerus|AA =_ *R*_Z_ (θ_Z_) ⋅ *P*_humerus∣AA +_ *T*_Y_​

This adjustment allowed simulation of acromial tilt and elevation while maintaining consistency with the scapula-based reference frame.

**Restoration**

When the glenoid was restored to its native orientation, the applied rotations were reversed using the transpose of the planned rotation matrix:

(6) *R*_Restored scapula∣glenoid =_ (R_X_ (θ_X_) ⋅ R_Y_ (θ_Y_)) ^T^ *⋅ R _new scapula∣glenoid_*

so that kinematics could again be expressed relative to the unaltered glenoid.

**Summary**

This workflow provides a systematic and ISB-compliant method for transforming humeral kinematic data from a scapula-based frame to the glenoid frame. By applying surgical plan rotations as active anatomical changes about the glenoid origin, the analysis ensures that humeral motion is consistently described with respect to the functional articular surface. These post-hoc transformations enable accurate assessment of glenohumeral motion across both baseline and surgically altered conditions.
